# A Note on “A Catalog of Biases in Questionnaires”

**Published:** 2005-03-15

**Authors:** Jeffrey J Harrow

**Affiliations:** Veterans Integrated Service Network (VISN) 8, Patient Safety Center of Inquiry, James A. Haley VA Medical Center, Tampa, Fla

## To the Editor:

The authors of "A Catalog of Biases in Questionnaires" in the January issue (1) provide a useful catalog of questionnaire biases, but in one of their examples, their recommended response is guilty of the same bias. This error occurs in Overlapping Interval.

*Example:*

*How many cigarettes do you smoke per day?*

[ ] None  [ ] 5 or less   [ ] 5–25    [ ] 25 or more

The authors' recommended question was:

*How many cigarettes do you smoke per day?*

[ ] None  [ ] 4 or less  [ ] 5–24    [ ] 25 or more

While this corrects the ambiguity of the first question for respondents who smoke exactly 5 or 25 cigarettes per day, it maintains the ambiguity for nonsmokers. The recommended question should be:

*How many cigarettes do you smoke per day?*

[ ] None   [ ] 1–4   [ ] 5–24   [ ] 25 or more

Read the author's reply
